# Prognostic impact of initial platelet count and post-induction platelet recovery in elderly AML patients: associated with circulating cytokines

**DOI:** 10.3389/fonc.2025.1534158

**Published:** 2025-02-26

**Authors:** Chun Ling, Neng-Neng Cao, Xiao-Wei Niu, Shi-Yun Xu, Wen-Yu Gong, Wen-Qiang Bao, Qi-Chuan Jin, Yin Wang, Jing Wu, Chang-Zhi Zhao, Wen-Jing Fu, Qi-Guo Zhang, Shan-Shan Feng, Dao-Yuan Li

**Affiliations:** ^1^ Department of Hematology, Affiliated Chuzhou Hospital of Anhui Medical University, The First People’s Hospital of Chuzhou, Chuzhou, Anhui, China; ^2^ Department of Hematology, Hematological Research Center, The Second Affiliated Hospital of Anhui Medical University, Hefei, Anhui, China; ^3^ Heart Center, The First Hospital of Lanzhou University, Lanzhou, Gansu, China; ^4^ Laboratory Medicine Center, Affiliated Chuzhou Hospital of Anhui Medical University, Chuzhou, Anhui, China

**Keywords:** acute myeloid leukemia, platelets, circulating cytokines, prognostic, geriatric

## Abstract

**Objective:**

Acute myeloid leukemia (AML) is a highly heterogeneous hematologic malignancy, with various clinical features influencing its prognosis. The aim of this study to evaluate the impact of platelet count at diagnosis and platelet recovery after induction chemotherapy on the survival outcomes of elderly AML patients.

**Methods:**

A total of 109 elderly patients with AML who were treated in our center between 2017 and 2023 were evaluated. According to the median platelet counts at the time of new diagnosis, the cases were divided into a low platelet counts group (≤40×10^9^/L, n=54) and a high platelet count group (>40×10^9^/L, n=55). Platelet recovery times were accepted as the periods from the beginning of induction chemotherapy to a platelet count of ≥20×10^9^/L 3 days in a row, respectively. The median time to platelet recovery was 25 days (range12-47) for all patients. Therefore, platelet recovery in the first 25 days was defined as early platelet recovery and at >25 days it was defined as late platelet recovery.

**Results:**

Low platelet counts at diagnosis and early recovery of platelet counts after induction therapy indicate longer overall survival (OS) and Leukemia-free survival (LFS). Patients with high platelet counts at diagnosis and those with delayed platelet recovery after induction therapy exhibited elevated levels of interleukin-1β (IL-1β) and tumor necrosis factor-α (TNF-α). Additionally, patients with high platelet counts at diagnosis also had relatively higher levels of interleukin-8 (IL-8).

**Conclusion:**

Platelets can be used as a prognostic biomarker for elderly AML and may be associated with circulating cytokines.

## 1 Introduction

Acute myeloid leukemia (AML) is a highly heterogeneous hematologic malignancy, with a median age of 68 years at diagnosis (1). Older age can be considered a poor prognostic factor for AML, making the treatment of elderly AML patients particularly challenging. In addition to the crucial roles of genetic and molecular abnormalities in risk stratification and prognosis assessment for AML, several clinical features are also vital, such as white blood cell count and central nervous system involvement. Compared to these prognostic factors, the significance of platelet count in the prognosis of elderly AML patients has not been fully evaluated.

Most elderly leukemia patients lose the opportunity for hematopoietic stem cell transplantation at the time of diagnosis, and the efficacy of chemotherapy largely determines their prognosis. Therefore, we aim to identify more clinical features to assess the prognosis of elderly AML patients. In this study, we monitored platelet counts at diagnosis and after induction chemotherapy in elderly AML patients and subsequently evaluated their significance in terms of general clinical characteristics, diagnostic risk stratification, induction therapy response, treatment maintenance, and survival. Given the immune-related properties of platelets, we also analyzed their association with cytokine profiles.

## 2 Patients and methods

### 2.1 Patients

**Figure 1:**
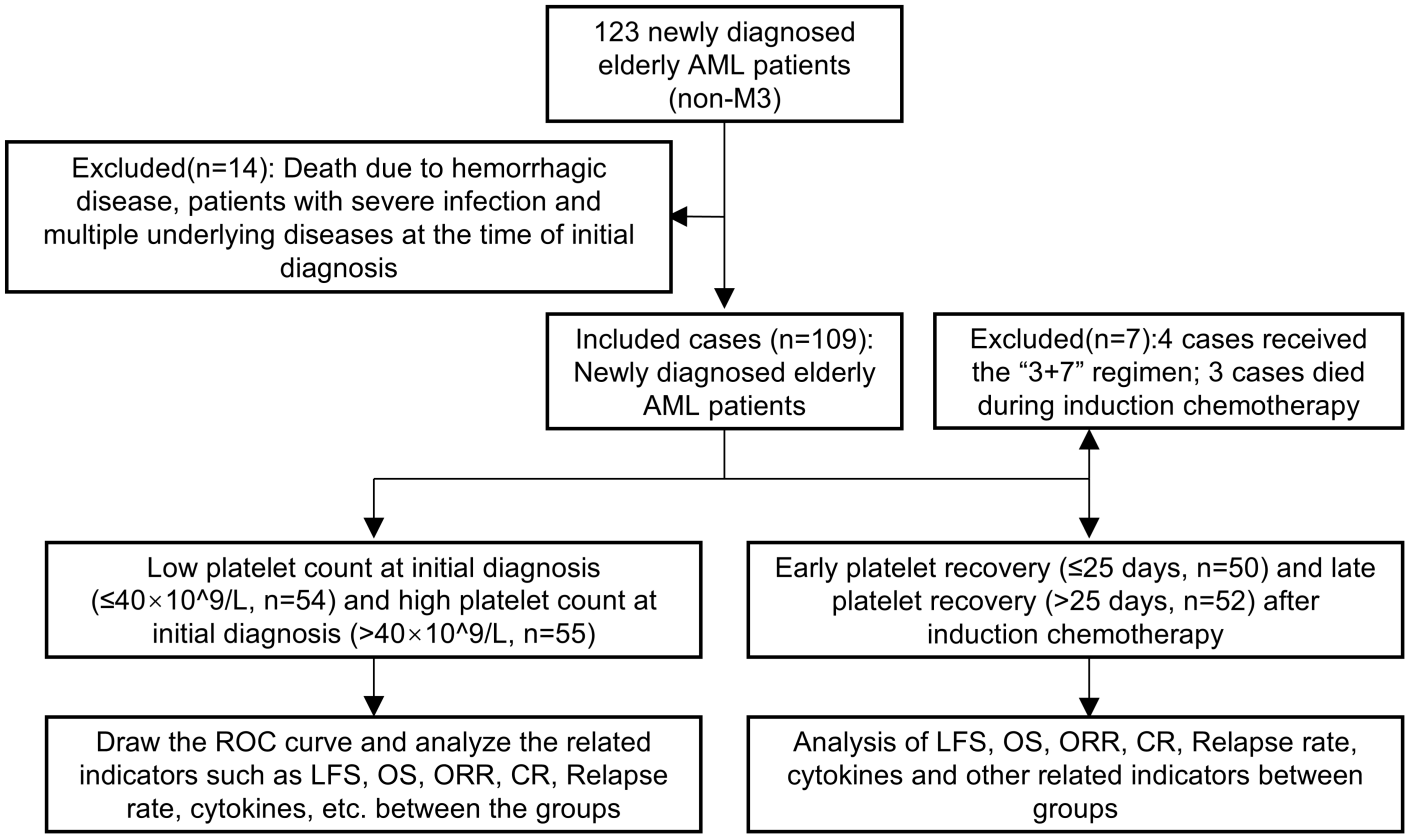
The selection of participants for analysis and the study flowchart.

This study was approved by the Institutional Review Board of Chuzhou Hospital, affiliated with Anhui Medical University. Between January 2017 and December 2023, our center admitted 123 newly diagnosed acute myeloid leukemia (AML) patients aged ≥60 years, excluding those with acute promyelocytic leukemia (APL). AML was classified based on the National Comprehensive Cancer Network (NCCN) Guidelines (2). Fourteen patients were excluded due to death from hemorrhagic disorders, severe infections at initial diagnosis, or multiple comorbidities, leaving 109 patients for inclusion in the study. Patients received 1–2 cycles of initial induction chemotherapy, followed by consolidation therapy according to risk stratification and relevant risk assessments. Patients who underwent allogeneic hematopoietic stem cell transplantation were excluded from this study. The detailed characteristics of the 109 elderly AML patients are summarized in **Table 1**, and the study flowchart is presented in **Figure 1**.

**Table 1:** Characteristics of 109 newly diagnosed elderly patients with AML.

Characteristics	Total (N = 109)		
	N	Median (range)	%
Age		70 (60-90)	
Sex			
Male	62		56.9
Female	47		43.1
FAB classification			
M0	2		1.8
M1	10		9.2
M2	55		50.5
M4	8		7.3
M5	28		25.7
M6	1		0.9
Mu	5		4.6
ELN2017 risk stratification			
Low-medium risk	43		39.4
High risk	62		56.9
not classifiable	4		3.7
Initial CBC			
WBC (10^9^/L)		2.27 (0.14-218.05)	
Hb(g/L)		76 (26-121)	
PLT (10^9^/L)		40 (2-399)	
LDH(IU/L)		224.3 (59.5-1665.3)	
ECOG PS			
0–2	90		82.6
≥3	19		17.4

FAB, France America and Britain; Mu, unclassifiable AML in morphology; ELN2017, European Leukemia Net 2017; WBC, white blood cell; Hb, hemoglobin; PLT, platelet; LDH, lactate dehydrogenase; ECOG PS, Eastern Cooperative Oncology Group Performance Status.

### 2.2 Treatment and evaluation

Induction chemotherapy followed the DA/IA (idarubicin, 8–12mg/m^2^/day1-3, or daunorubicin, 45–60mg/m^2^/day1-3; and cytarabine,100 mg/m^2^/day1-7) regimen(n=4). Patients who are unable to tolerate high-intensity induction regimens were given a D-CAG (Decitabine,15 mg/m^2^/day1-5; and granulocyte colony stimulating factor of 300 μg/day0-9 for priming in combination with cytarabine 10mg/m^2^/q12h day3-9 and aclarubicin 10 mg/day 3–6) regimen as induction therapy(n=105). Patients who did not achieve CR or partial remission (PR) were offered alternative therapies. Post-remission therapy consisted of 4–6 cycles D-CAG or conventional chemotherapy. Bone marrow examination was performed 2 weeks after the end of induction therapy, and efficacy was evaluated according to the NCCN guidelines (2).

### 2.3 Platelet counts analysis

The platelet counts of the 109 patients with AML at diagnosis were obtained from routine blood examinations recorded in the clinical laboratory records, determined by the Sysmex XN1000 hematology analyzer (Sysmex Corporation, Kobe).

### 2.4 Immunomodulatory circulating cytokines analysis

Cytokines were measured by EasyMagPlex Human Cytokine 12 Plex Kit (Shenzhen Wellgrow Technology Inc.) in the department of clinical laboratory. The panel of measured cytokines included IL-1β, IL-2, IL-4, IL-5, IL-6, IL-8, IL-10, IL-12p70, IL-17, interferon (IFN)-α, IFN-γ, and TNF-α (3).

### 2.5 Statistical analysis

Statistical analyses were performed using SPSS 21.0 and GraphPad Prism 8 software. Quantitative data were expressed as mean ± SD. The chi-square test and Student’s t-test were used to evaluate the significance of differences between groups. When variance within groups was unequal, the non-parametric Mann-Whitney test was applied. Prognostic value was assessed using Kaplan-Meier survival curves. Overall survival (OS) was defined as the time from diagnosis to the date of death. Leukemia-free survival (LFS) was defined as the time from complete remission (CR) to the first relapse. A p-value less than 0.05 was considered statistically significant.

## 3 Results

### 3.1 Patient characteristics

From January 2017 to December 2023, 109 newly diagnosed elderly AML patients were enrolled in this study. Patients diagnosed with acute promyelocytic leukemia (APL) and those who died from hemorrhagic events before the completion of induction therapy were excluded. The median platelet count at diagnosis was 40.00×10^9^/L, ranging from 2.0×10^9^/L to 399.00×10^9^/L. Patients with platelet counts less than or equal to the median (≤40.00×10^9^/L) were classified as the low platelet count group (n=54), while those with platelet counts greater than the median (>40.00×10^9^/L) were classified as the high platelet count group (n=55). Platelet recovery time was defined as the time from the start of induction chemotherapy to the point when the platelet count remained ≥20×10^9^/L for three consecutive days without transfusion support. The median time to platelet recovery for all patients was 25 days (range=12-47). Early platelet recovery was defined as recovery within 25 days, while late platelet recovery was defined as ≥26 days.

There were no statistically significant differences between the low platelet counts group and the high platelet counts group in terms of age, gender, risk stratification, lactate dehydrogenase (LDH) levels, hemoglobin levels, and ECOG performance status (P > 0.05). However, a statistically significant difference was observed in white blood cell count levels between the two groups (P < 0.05) (**Table 2**).

**Table 2:** Clinical characteristics of platelet count at initial diagnosis and platelet recovery after induction chemotherapy in elderly patients with AML.

Characteristics	PLT>40×10^9^/L (N=55)		PLT ≤ 40×10^9^/L (N=54)		*P*	Early platelet recovery (N=50)		Late platelet recovery (N=52)		*P*
	N	Mean ± SD	N	Mean ± SD		N	Mean ± SD	N	Mean ± SD	
Age(years)		70.04 ± 7.53		70.24 ± 7.46	0.8895		69.34 ± 6.64		71.21 ± 7.99	0.2025
Sex					0.6193					0.3043
Male	30		32			31		27		
Female	25		22			19		25		
ELN2017 risk stratification					0.283					<0.001
Low-medium risk	19		24			34		9		
High risk	34		28			16		41		
Not classifiable	2		2			0		2		
WBC (10^9^/L)		22.30 ± 49.59		7.50 ± 21.73	0.0468		6.99 ± 20.05		24.31 ± 50.71	0.0265
Hb(g/L)		77.69 ± 17.81		76.13 ± 16.74	0.6386		78.86 ± 17.20		75.42 ± 17.56	0.3202
LDH(IU/L)		321.33 ± 248.06		285.69 ± 273.55	0.4775		249.49 ± 152.91		367.12 ± 334.59	0.0255
ECOG PS					0.4229					<0.001
0–2	47		43			49		37		
≥3	8		11			1		15		

ELN2017, European Leukemia Net 2017; WBC, white blood cell; Hb, hemoglobin; PLT, platelet; LDH, lactate dehydrogenase; ECOG PS, Eastern Cooperative Oncology Group Performance Status.

Additionally, there were no statistically significant differences between the early and late platelet recovery groups in terms of age, gender, and hemoglobin levels (P > 0.05). However, significant differences were found in white blood cell count and lactate dehydrogenase levels (P < 0.05), as well as in risk stratification and ECOG performance status (P < 0.001) (**Table 2**).

### 3.2 High platelet counts and late platelet recovery predict shorter duration of remission and survival in elderly AML patients

There were no statistically significant differences between the low platelet count group and the high platelet count group in terms of overall response rate (ORR), complete remission (CR) rate, and relapse rate (P > 0.05), but a significant difference was observed in the continuous complete remission (CCR) rate (P < 0.05). However, there were significant statistical differences between the early platelet recovery group and the late platelet recovery group in overall response rate, CR rate, CCR rate, as well as in early and late relapse rates (P < 0.01) (**Table 3**).

**Table 3:** Comparison of therapeutic effects among groups with different platelet counts and recovery periods.

	PLT>40×10^9^/L (N=55)	PLT ≤ 40×10^9^/L (N=54)	*P*	Early platelet recovery (N=50)	Late platelet recovery (N=52)	*P*
	N (%)	N (%)		N (%)	N (%)	
ORR	46 (83.64)	47 (87.04)	0.6160	50 (100.00)	43 (82.69)	0.0021
CR	43 (78.18)	43 (79.63)	0.6461	50 (100.00)	36 (69.23)	0.0009
NR	9 (16.36)	7 (12.96)		0 (0)	9 (17.31)	
CCR	7 (12.73)	20 (37.04)	0.0024	26 (52.00)	1 (1.92)	<0.0001
Relapse	35 (63.64)	22 (40.74)		22 (44.00)	35 (67.31)	
Early relapse	22 (40.00)	9 (16.67)	0.1053	9 (18.00)	26 (50.00)	0.0017
Late relapse	13 (23.64)	13 (24.07)		13 (26.00)	9 (17.31)	

ORR overall response rate, CR complete remission, NR non-response after two induction chemotherapy, refers to refractory, CCR continuous CR.

### 3.3 The platelet count at initial diagnosis can reliably predict prognosis and survival in elderly AML patients

**Figure 2:**
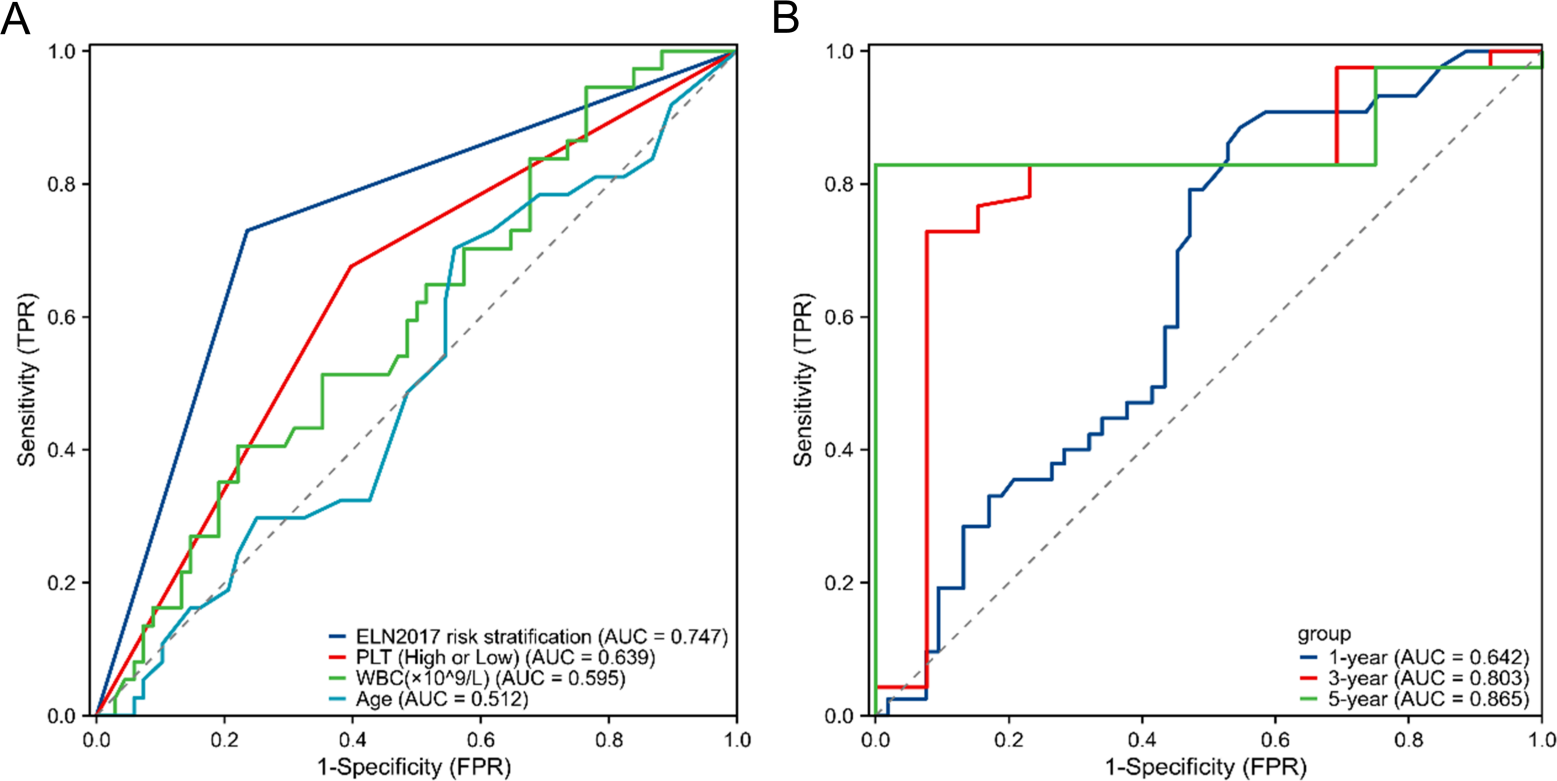
ROC curve for the prediction of prognosis and survival of elderly AML. **(A)** ROC curve for survival prediction using the platelet count at initial diagnosis and other variables (ELN 2017 risk stratification, age, WBC). **(B)** Alidation of the predictive efficiency of the platelet count at initial diagnosis through ROC curve analysis. ROC, receiver operating characteristic; AML, acute myeloid leukemia; WBC, white blood cell; ELN2017, European Leukemia Net 2017.

The ROC curve analysis indicates that the platelet count at initial diagnosis has a certain advantage in predicting the prognosis of elderly AML patients. Although this advantage is not as strong as the ELN 2017 risk stratification, it is significantly better than white blood cell counts and age (**Figure 2A**). Time-dependent ROC curve analysis showed that the accuracy for predicting 1-, 3-, and 5-year OS was 0.64238 (95% CI 0.5316-0.7532), 0.80334 (95% CI 0.6607-0.946), and 0.86541 (95% CI 0.7777-0.9532), respectively. These results suggest that the platelet count at initial diagnosis can be a relatively accurate predictor of survival in elderly AML patients (**Figure 2B**).

### 3.4 High platelet counts and late platelet recovery predict poorer OS and LFS in elderly AML patients

**Figure 3:**
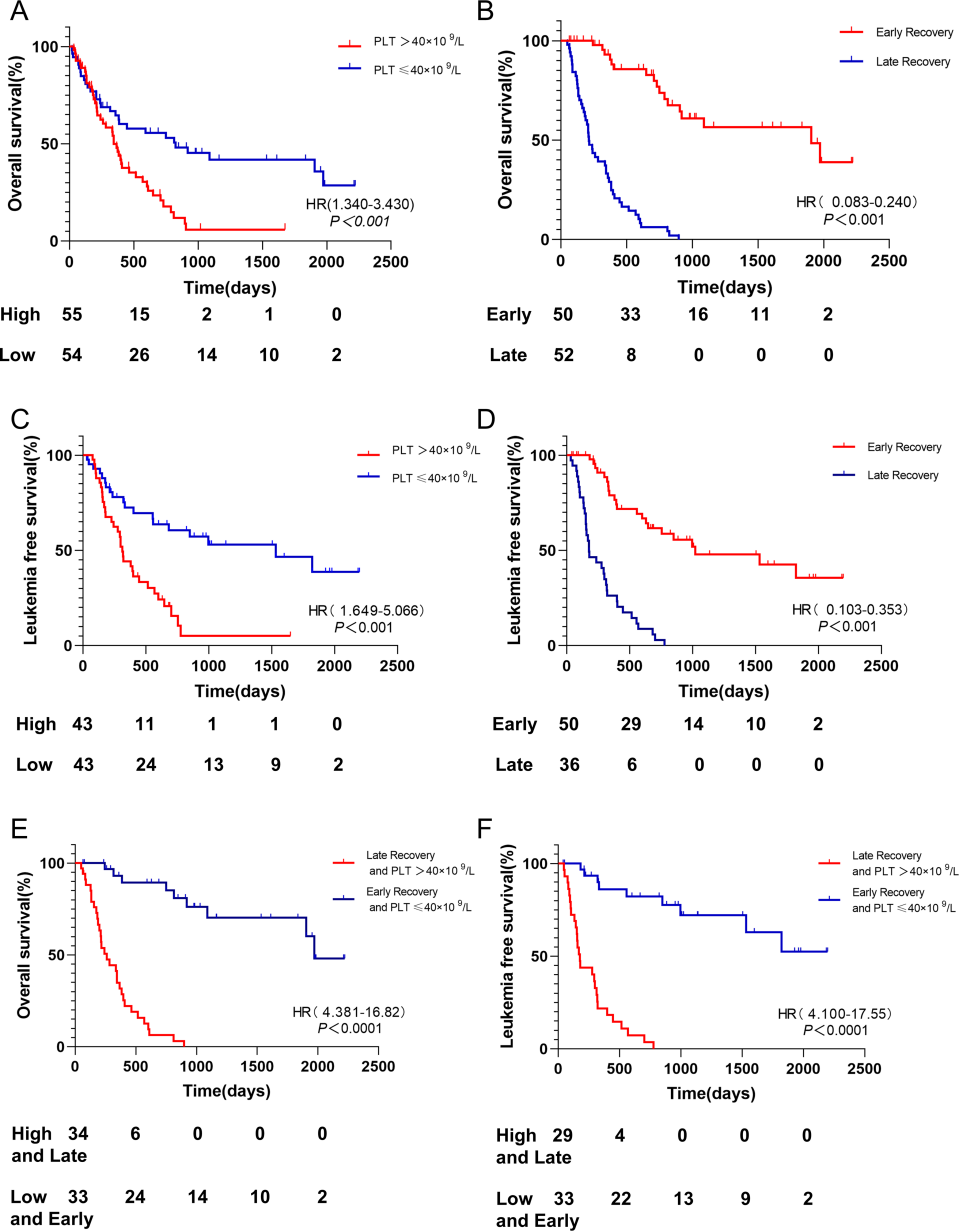
OS and LFS among different groups. **(A)** Patients with platelet count ≤40 × 10^9^/L had longer OS than PLT > 40 × 10^9^/L. **(B)** Patients with early platelet recovery after induction chemotherapy had longer OS than those with late platelet recovery. **(C)** Patients with platelet count ≤40 × 10^9^/L had longer LFS than PLT > 40 × 10^9^/L. **(D)** Patients with early platelet recovery after induction chemotherapy had longer LFS than those with late platelet recovery. **(E)** Patients with platelet count ≤40 × 10^9^/L and early platelet recovery had longer OS than PLT > 40 × 10^9^/L and late platelet recovery. **(F)** Patients with platelet count ≤40 × 10^9^/L and early platelet recovery had longer LFS than PLT > 40 × 10^9^/L and late platelet recovery. OS, Overall Survival; PLT platelet; LFS, leukemia-free survival.

In elderly AML patients, those with high platelet counts at initial diagnosis had a shorter OS compared to those with low platelet counts (median survival: 345 days vs 825 days, p < 0.001, **Figure 3A**). Similarly, patients with late platelet recovery had significantly shorter OS compared to those with early platelet recovery (median survival: 214 days vs 1905 days, p < 0.001, **Figure 3B**).

Moreover, patients with high platelet counts at initial diagnosis had a shorter LFS compared to those with low platelet counts (median survival: 313 days vs 996 days, p < 0.001, **Figure 3C**). Patients with late platelet recovery also had significantly shorter LFS compared to those with early platelet recovery (median survival: 177 days vs 1021 days, p < 0.001, **Figure 3D**).

Patients with low platelet counts at initial diagnosis and early platelet recovery had a significantly better OS compared to those with high platelet counts at initial diagnosis and delayed platelet recovery (median survival: 1971 days vs 260 days, p < 0.001, **Figure 3E**). The median survival of LFS was not reached in patients with low platelet counts at initial diagnosis and early platelet recovery, while it was 177 days in those with high platelet counts at initial diagnosis and late platelet recovery (p < 0.001, **Figure 3F**).

### 3.5 High platelet counts at initial diagnosis and late platelet recovery in elderly AML patients are associated with immunoregulatory cytokines

**Figure 4:**
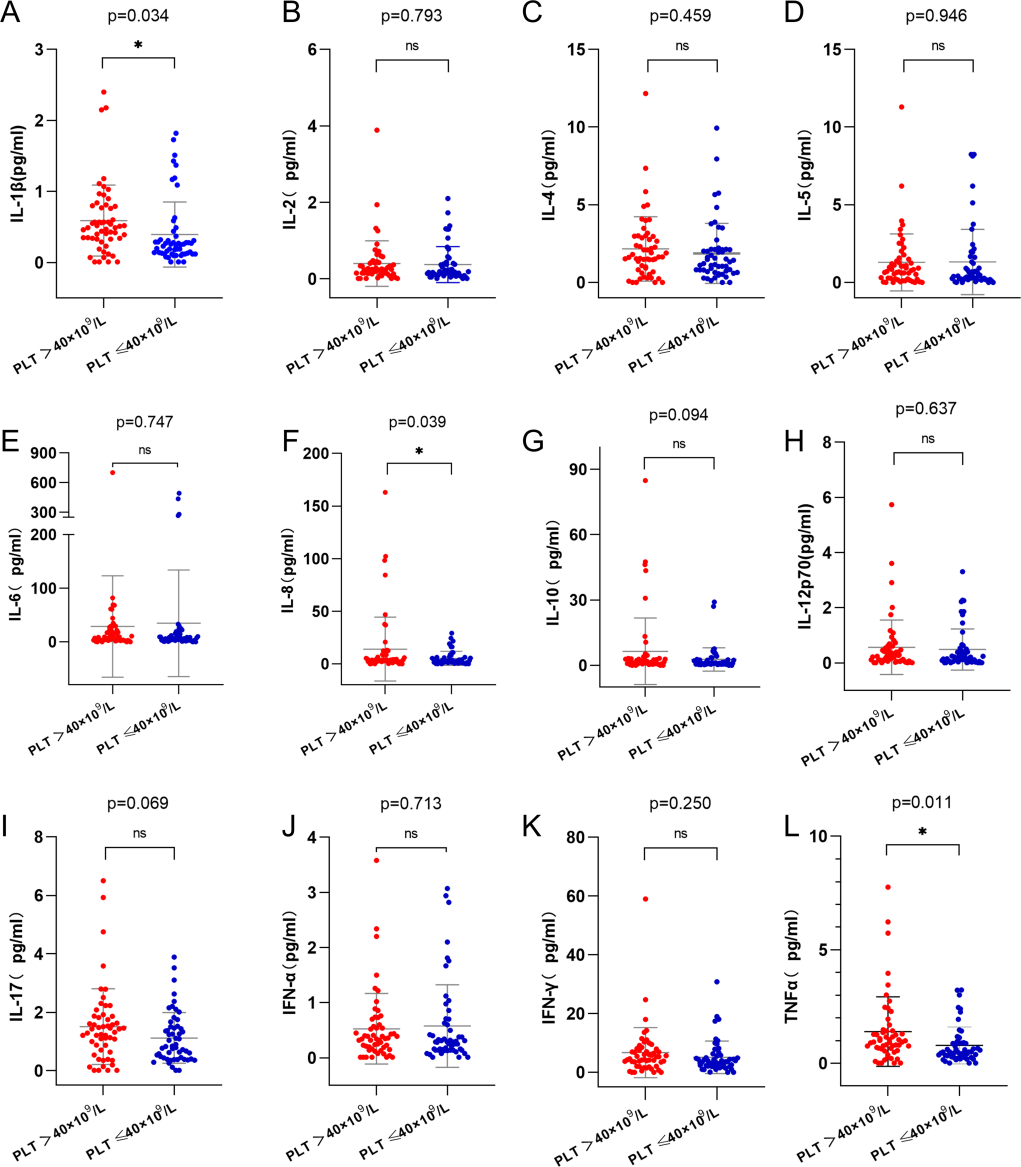
Analysis of cytokines in high and low platelet counts groups. **(A)** IL-1β level in the high platelet counts group was higher than that in the low platelet counts group (p=0.034). **(F)** IL-8 level in the high platelet counts group was higher than that in the low platelet counts group(p=0.039). **(L)** TNF-α level in the high platelet counts group was higher than that in the low platelet counts group (p=0.011). **(B-E, G-K)**, there was no statistically significant difference in the levels of cytokines between the two groups(p>0.05). IL-1β, Interleukin-1β; IL-8, Interleukin-8; TNF-α, Tumor necrosis factor-α. ns, *p*>0.05; *, *p*>0.05.

To investigate the relationship between platelet counts at initial diagnosis, post-induction platelet recovery, and plasma cytokines, we measured 12 plasma cytokines, including IL-1β, IL-2, IL-4, IL-5, IL-6, IL-8, IL-10, IL-12p70, IL-17, IFN-α, IFN-γ and TNF-α. The results showed that levels of IL-1β, IL-8, and TNF-α were significantly higher in the high platelet count group compared to the low platelet count group (**Figure 4A**, p=0.034; **Figure 4F**, p=0.039; **Figure 4L**, p=0.011). No significant differences were observed between the two groups in the levels of IL-2, IL-4, IL-5, IL-6, IL-10, IL-12p70, IL-17, IFN-α, or IFN-γ (**Figures 4B-E, G-K,** p>0.05).

**Figure 5:**
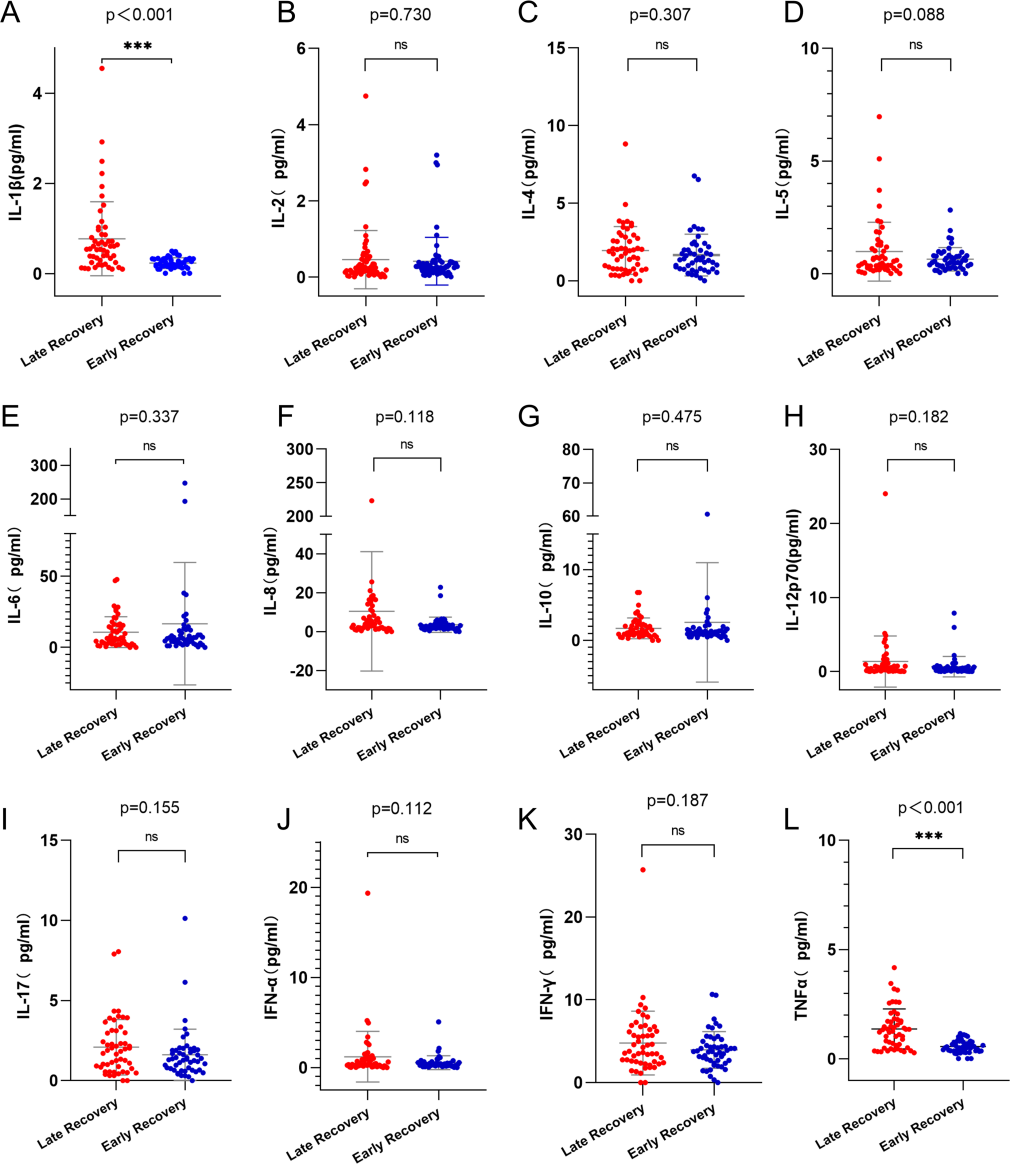
Analysis of cytokines in early and late platelet recovery groups. **(A)** IL-1β level in the late platelet recovery group was higher than that in the early platelet recovery group (p<0.001). **(L)** TNF-α level in the late platelet recovery group was higher than that in the early platelet recovery group (p<0.001). **(B-K)** there was no statistically significant difference in the levels of cytokines between the two groups(p>0.05). IL-1β, Interleukin-1β; TNF-α, Tumor necrosis factor-α. ns, *p*>0.05; ***, *p*>0.001.

After induction chemotherapy, levels of IL-1β and TNF-α were significantly higher in the late platelet recovery group compared to the early platelet recovery group (**Figure 5A**, p < 0.001; **Figure 5L**, p < 0.001). There were no statistically significant differences between the two groups in the levels of IL-2, IL-4, IL-5, IL-6, IL-8, IL-10, IL-12p70, IL-17, IFN-α or IFN-γ (**Figures 5B–K**, p > 0.05).

## 4 Discussion

Advanced age is a well-established adverse prognostic factor in AML, making clinical management of this patient population particularly challenging. High white blood cell count at diagnosis is also recognized as a high-risk factor in AML, often associated with poor prognosis and shorter survival times. However, the prognostic significance of platelet count in AML patients remains a topic of ongoing debate. One study found that AML patients with an initial platelet count in the range of 50-120×10^9^/L had a better prognosis compared to others (4), while another study indicated that patients with an initial platelet count of ≤40×10^9^/L had a more favorable prognosis in intermediate-risk AML (5). Some studies have also indicated that AML patients with a high platelet count at diagnosis tend to have a poorer prognosis and shorter survival time (6, 7). Additionally, platelet recovery after induction chemotherapy can also be a relatively accurate predictor of prognosis in AML patients. Some studies have shown that early platelet recovery after induction chemotherapy is associated with a relatively better prognosis (8, 9). The above examples are based on studies of the general adult population, but similar research specifically focused on elderly AML patients is lacking. Therefore, we conducted a single-center study aimed at elucidating the impact of initial platelet count and platelet recovery after induction chemotherapy on the prognosis of elderly AML patients.

Our study revealed that elderly AML patients with high initial platelet counts and late platelet recovery after induction chemotherapy have significantly worse outcomes, including shorter OS and LFS, compared to those with low initial platelet counts and early platelet recovery. These findings suggest that both initial platelet counts and the timing of platelet recovery post-chemotherapy can serve as important prognostic indicators in elderly AML patients.

In addition to platelet count at diagnosis, the timing of platelet recovery following induction chemotherapy also emerged as a critical prognostic factor. Patients who exhibited late platelet recovery had markedly shorter OS and LFS compared to those with early platelet recovery. This suggests that late platelet recovery might reflect an underlying resistance to chemotherapy or a more extensive disease state that hampers hematopoietic recovery (10–12). This finding is particularly relevant for elderly AML patients, who often have compromised bone marrow reserves and a diminished capacity for hematopoietic regeneration, making them more susceptible to prolonged cytopenia and associated complications.

We also explored the relationship between platelet counts and cytokine levels, shedding light on the potential mechanisms underlying the observed prognostic differences. We found that patients with high initial platelet counts had significantly elevated levels of pro-inflammatory cytokines, including IL-1β, IL-8, and TNF-α, compared to those with low platelet counts. These cytokines are known to play crucial roles in the inflammatory response (13) and have been implicated in the pathophysiology of various malignancies, including AML (14). The elevated cytokine levels in the high platelet count group suggest a more pronounced inflammatory milieu, which could contribute to disease progression and resistance to therapy (15).

Similarly, patients with late platelet recovery also exhibited higher levels of IL-1β and TNF-α compared to those with early recovery. This suggests that persistent inflammation may be a contributing factor to delayed hematopoietic recovery and, consequently, poorer outcomes (16). The link between inflammation and impaired hematopoiesis is well-documented, with chronic inflammatory states known to disrupt normal bone marrow function and promote leukemic cell survival (17). Therefore, the elevated cytokine levels observed in the late platelet recovery group may reflect an ongoing inflammatory process that hinders effective hematopoietic recovery and contributes to the adverse prognosis.

These findings underscore the importance of considering platelet counts and recovery kinetics as part of the overall prognostic assessment in elderly AML patients. The association between high platelet counts, late platelet recovery, and elevated cytokine levels suggests that these factors may be interconnected, potentially reflecting a common underlying pathophysiological process involving inflammation and impaired hematopoiesis. This could have important implications for the management of elderly AML patients, as it highlights the need for tailored therapeutic approaches that address not only the leukemic burden but also the associated inflammatory state.

Moreover, the identification of high platelet counts and late platelet recovery as adverse prognostic factors could inform risk stratification and treatment decisions in this patient population. For instance, patients with these characteristics might benefit from more aggressive or targeted therapies aimed at modulating the inflammatory response or enhancing hematopoietic recovery. Conversely, the relatively favorable prognosis associated with low platelet counts and early recovery could guide clinicians towards less intensive treatment regimens, thereby minimizing toxicity while still achieving effective disease control.

## 5 Conclusions

Our study provides new insights into the prognostic significance of platelet counts and recovery dynamics in elderly AML patients. The findings suggest that high initial platelet counts and delayed recovery after induction chemotherapy are associated with poorer outcomes and there is a correlation with elevated cytokine levels, potentially due to an underlying inflammatory state that impairs hematopoietic recovery and promotes leukemic cell survival. These results emphasize the need for further research to explore the mechanisms linking platelet counts, cytokine levels, and AML prognosis, with the ultimate goal of improving risk stratification and treatment strategies for this vulnerable patient population.

## Data Availability

The raw data supporting the conclusions of this article will be made available by the authors, without undue reservation.
